# Submerged Osmotic Processes: Design and Operation to Mitigate Mass Transfer Limitations

**DOI:** 10.3390/membranes8030072

**Published:** 2018-09-01

**Authors:** Gaetan Blandin, Ignasi Rodriguez-Roda, Joaquim Comas

**Affiliations:** 1 Laboratory of Chemical and Environmental Engineering (LEQUIA), Institute of the Environment, University of Girona, 17003 Girona, Spain; irodriguezroda@icra.cat (I.R.-R.); joaquim.comas@udg.edu (J.C.); 2 Catalan Institute for Water Research (ICRA), 17003 Girona, Spain

**Keywords:** membrane bioreactor, forward osmosis, water reuse, submerged membrane, concentration polarization, module design, vacuum assisted osmosis

## Abstract

Submerged forward osmosis (FO) is of high interest for bioreactors, such as osmotic membrane bioreactor, microalgae photobioreactor, food or bioproduct concentration where pumping through pressurized modules is a limitation due to viscosity or breakage of fragile components. However, so far, most FO efforts have been put towards cross flow configurations. This study provides, for the first time, insights on mass transfer limitations in the operation of submerged osmotic systems and offer recommendations for optimized design and operation. It is demonstrated that operation of the submerged plate and frame FO module requires draw circulation in the vacuum mode (vacuum assisted osmosis) that is in favor of the permeation flux. However, high pressure drops and dead zones occurring in classical U-shape FO draw channel strongly disadvantage this design; straight channel design proves to be more effective. External concentration polarization (ECP) is also a crucial element in the submerged FO process since mixing of the feed solution is not as optimized as in the cross flow module unless applying intense stirring. Among the mitigation techniques tested, air scouring proves to be more efficient than feed solution circulation. However, ECP mitigation methodology has to be adapted to application specificities with regards to combined/synergetic effects with fouling mitigation.

## 1. Introduction

**Figure 1:**
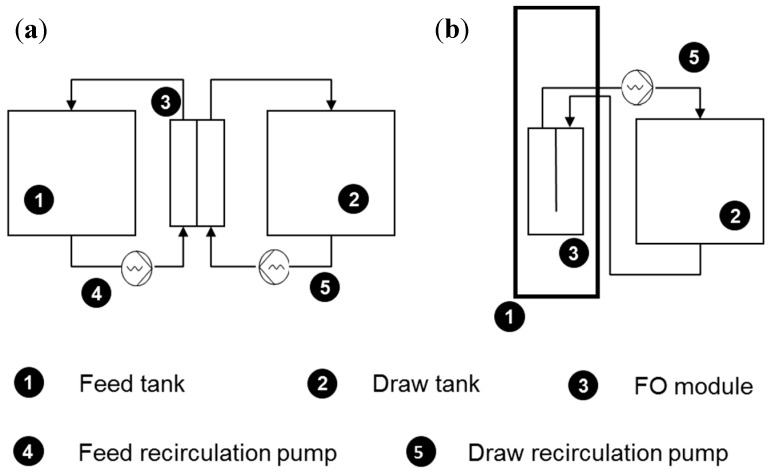
Schematics of a cross flow (**a**) and a submerged FO (**b**) systems.

Osmotic processes, such as forward osmosis (FO) and pressure retarded osmosis (PRO), have gained interests for the last two decades, following the commercialization of the first dedicated FO membrane by HTI (Albany, OR, USA) [1,2]. Intense development leads to further progress towards the improvement of membrane properties, draw solutions and better identifying applications [3,4]. More fundamental research is also dedicated to the better understanding of mechanisms related to mass transfer limitation, rejections of micropollutants and fouling [4,5,6,7,8,9,10,11]. However, so far, most studies have been performed using membrane coupons in a cross flow cell (CFC) [12]. The CFC allows for membrane characterization and study of fundamental aspects in controlled conditions, but results cannot directly be transferred to performances in applications and with regards to various process configurations. Moving towards large-scale applications, few studies evaluated other aspects of FO/PRO usage through module scale operation and using different types of configurations (plate and frame (P&F), spiral wound, hollow fiber) but always in a cross flow configuration ([13,14,15,16,17,18,19], Figure 1a). As such, so far, osmotic processes have been mostly studied in systems, whereby feed and draw solutions are pumped through a chamber/module, where the membrane filtration occurs. The FO, due to its specific features, allows for rejection of most components, such as reverse osmosis (RO), but unlike the RO, the FO does not rely on hydraulic pressure. Due to high pressure operation, limiting design restriction, therefore, no longer stands; with the FO, both new applications and novel module configurations, such as submerged modules, can be envisioned. In the latter case (Figure 1b), the FO module is submerged in a (feed) tank, and consequently, very distinct operation from a closed (pressurized) module is required.

As for submerged microfiltration/ultrafiltration bioreactors, submerged FO operation could be of high interest, owing to its limited shear stress for applications, such as cultivation of bioproducts, and concentrations to avoid breakage of fragile biological compounds [20]. As part of these applications, cultivation and separation of microalgae is of particular interest. Several studies evaluated the fouling of microalgae in the FO process but always in the CFC [21,22]; a very ambitious and innovative NASA project (OMEGA) cultivated microalgae within an FO membrane used as a tubular photobioreactor placed at the surface of seawater, but published data remain lab-scale using HTI X-Pack Hydration bags [23]. Another example of growing FO applications is food concentration, which takes benefits of low hydraulic pressure and temperature to keep organoleptic properties of food with low fouling tendency, and high solids content processing capability [24,25]. However, most tests were performed in the cross flow mode (cells and modules); submerged operation can help to achieve higher concentrations whenever pumping through modules is a limiting factor due to viscosity. So far, submerged FO operation has been tested only for the separation of sludge, and more specifically, acts as an alternative to the membrane bioreactor (MBR), called osmotic MBR (OMBR) that allows for production of high-quality water to promote its further reuse [26,27,28,29,30,31,32,33]. However, in most of these cases, very low permeation flux is obtained and no specific attention is paid to module design and operation [29].

MBRs, as a widely used mature technology, is usually adopted in submerged membrane systems; therefore, a large amount of studies have been published regarding MBR operation and optimization [34]. The main mass transfer limitation in MBR enabled systems is fouling, and therefore, most studies are conducted towards better understanding of fouling mechanisms and its mitigation in operation. Among the tested strategies, mechanical vibration [35,36] and scouring with air or biogas (for anaerobic MBRs) [37,38,39] were evaluated. Module design, energy demand and improved control are also all critical aspects that are assessed to optimize MBR operation [38,40,41,42]. If experience gained from MBR is crucial for FO submerged systems, a direct transposition cannot be expected due to differences in membrane types and driving forces. In an MBR, porous ultrafiltration (UF) or microfiltration (MF) membranes are used; they differ largely from FO membranes, since salts are not rejected with UF/MF membranes [32]. As such, external concentration polarization (ECP) does not occur in the MBR (unless ions are trapped in the fouling layer [36]), while it has proven to be of importance in FO, especially with the new generation of FO membranes featuring higher permeation flux [43,44]. This is a critical point to study, since in submerged osmotic system, mitigating ECP via the promotion of turbulences on the submerged/feed side cannot be easily realized through the help of spacers in cross flow systems. Another critical difference between the FO and MBR is the difference of driving force. In the MBR, the transmembrane pressure applied under slight vacuum conditions allows for homogeneous driving force through all the membrane filtration surface area and does not require complex design of the permeate side line. In the submerged FO, the draw solution needs to be circulated in such a way that it allows for an optimized distribution within the module and avoid dead zones [45]. The impact of draw channel design in the submerged FO module has not been studied so far.

The aim of this work was to bring better understanding on mass transfer limitations in the operation of submerged osmotic systems and to offer recommendations for optimized design and operation. At first, evidence of non-optimized mass transfer limitation of our formerly developed submerged module [46] versus the FO cross flow cell was demonstrated, and differences between both systems were listed to be specifically and systematically addressed. Then, with regards to draw side limitations, the impact of channel design for draw solution circulation (shape and type of spacers) and associated operating parameters (i.e., operating pressure, cross flow velocity (CFV), permeation flux and pressure) were elucidated. Evidences of feed side limitations associated with ECP (feed salinity and lack of turbulences at the membrane surface) were demonstrated, and mitigation techniques to promote turbulences were assessed (mechanical stirring, air scouring, feed circulation).

## 2. Materials and Methods

### 2.1. Membranes and Modules

**Figure 2:**
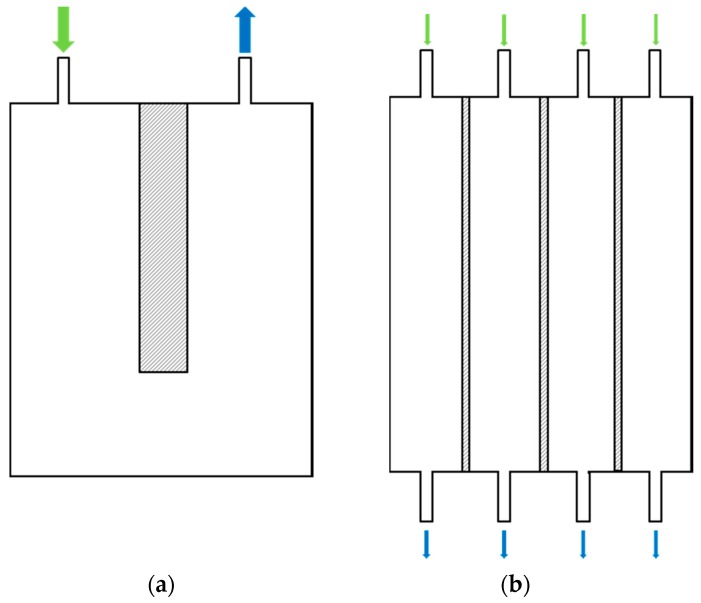
U-shape (**a**,**b**) straight (draw circulation) channels submerged FO plate designs. Draw inlet in green, draw outlet in blue.

A new generation of thin-film composite (TFC) commercially available FO membranes obtained from Toray Industries (Seoul, South Korea) membranes was used [14]. Home-made submerged FO plates were designed and realized based on Kubota MF cartridges 203. In order to evaluate the impact of draw channel design on submerged FO module performances, 3 specific designs were tested:

- U-shape large-spacer module: U-shape draw channel design (as commonly found in spiral-wound FO modules [3,47] with draw channel spacers containing a 1.2-mm-thickness diamond-type polypropylene mesh spacer composed of two levels of filaments to promote turbulences [48] (Figure 2a).
- U-shape thin-spacer module: U-shape draw channel design with draw channel spacers containing a 0.76-mm-thickness diamond-type polypropylene mesh (Figure 2a).
- Straight large-mesh spacer module: composed of 4 parallel channels containing a 1.2-mm-thickness diamond-type polypropylene mesh spacer (Figure 2b).

A draw solution was circulated under negative pressure pumping conditions as required for the submerged plate [29]. The water and salt fluxes obtained with the developed plates in the submerged mode were compared with those of similar membranes coupons in a CFC to evaluate the potential loss of performance associated with the module developed. The surface areas of membrane plates were 0.05 m^2^ (only one side of each plate was covered by membranes); plates were realized with a membrane active layer facing the feed solution.

### 2.2. Cross Flow Cell Setup

Membrane coupons were first evaluated in a CFC to evaluate membrane performances in optimized hydrodynamic conditions. Similar setups and operating conditions as in our former studies [49,50] were used with a 1.2-mm-thickness diamond-type polypropylene mesh spacer in both feed and draw channels and with a membrane active layer facing the feed solution. In the first set of tests, the membrane permeation flux was assessed using deionized water (DI) as feed and 10, 20, 35 and 70 g L^−1^ synthetic seawater (SW) as draw. Seasalts (>99.4% NaCl) was provided by Vicens i Batllori S.L. (Banyoles, Spain). Then, the effect of pumping was assessed; i.e., positive (pulsion) and negative (suction or vacuum) pumping, were performed by placing pump(s) before or after the CFC, respectively. These tests were realized for a range of flow rate varying from 0.12 to 0.36 L·min^−1^ (Table 1) for both feed and draw sides in order to assess the impact on pressure balance and induced permeation. Tests were performed using DI water as feed and DI water or 35 g·L^−1^ SW as draw solutions. All presented fluxes were average values of two tests. The water flux crossing the membrane from the feed to the draw was determined by measuring the increase of mass of the draw solution over time using a Kern PCB balance (Balingen, Germany) connected to a Bluetooth-RS232 Adaptor. Bluetooth based pressure sensors (0–4 bar) (Transducers Direct, Cincinnati, OH, USA) were used to record pressure in feed and draw channels. All instruments (pressure sensors and balances) data were recorded using the Bluetooth based system provided by Instrument Works (Waterloo, Australia).

### 2.3. Submerged Plate Lab Setup

The submerged FO plate was evaluated in the submerged mode in a tank with the following dimensions (length, 360 mm; width 560 mm; height, 260 mm). The plate was submerged in 20 L of DI water (feed) and 35 g·L^−1^ SW was circulated in the draw channel under negative pressure pumping (suction). The plate was maintained at the bottom of the tank with the membrane facing the top. The water in the tank (feed) was stirred vigorously using a vertical stirrer. The same system as for the CFC setup was used for water flux and data collection. Comparative evaluations were performed with and without stirring. In addition, as for the CFC, the permeation flux was assessed using DI water as feed and 10, 20, 35 and 70 g·L^−1^ SW as draw; then pressure drop was assessed for a range of flow rate varying from 0.12 to 0.48 L·min^−1^ (Table 1).

### 2.4. Submerged Plate Pilot Setup

**Figure 3:**
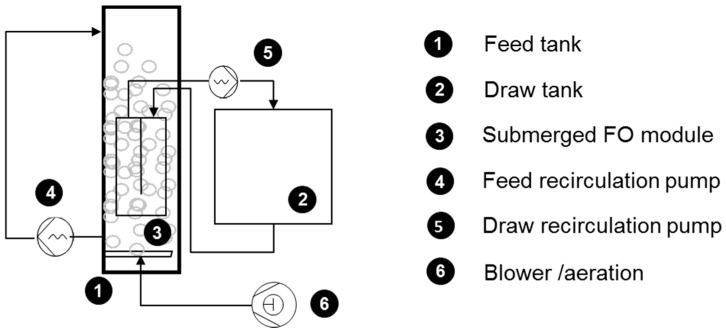
Submerged plate pilot setup.

Finally, in order to assess the impact of aeration and of feed recirculation in the submerged system, FO plates were placed in a stack fitting 3 plates that was submerged in a 25-L vertical reactor (length, 99 mm; width, 282 mm; height, 900 mm). Only one FO plate (0.05 m^2^ in area) was operated as before; the other two plates were used to mimic stack operation. 35 g·L^−1^ SW was circulated in the draw channel at a pump flow rate of 0.3 L·min^−1^ under negative pressure pumping (Figure 3). Aeration was provided below the FO plates through 4 diffusers, and the aeration rate in the range of 200 to 1000 L·h^−1^ (0.67 to 3.33 m^3^·m^−2^·h^−1^) was tested. Feed recirculation was provided by a peristaltic pump by pumping out water from the bottom of the reactor and pumping in water at the top. A typical feed recirculation rate of 0.3 L.min^−1^, which was the same as applied in OMBR operation in our former study, was initially tested [46]. However, given the minimal corresponding CFV (i.e., 0.4 × 10^−4^ m·s^−1^), additional tests were performed by limiting the cross section of the reactor and increasing the pump speed to operate with a recirculation rate up to the observed rate in microalgae cultivation raceway ponds, i.e., 0.1 m·s^−1^.

### 2.5. Water Flux Modelling

The theoretical water flux has been calculated based on existing solution diffusion models developed for FO by using Equation (1) as described in [44] to account for hydraulic pressure. Theoretical flux was then compared to experimental values to assess the impact of pumping conditions (negative or positive pressure pumping, cross flow velocity) on pressure balance in the system and its impact on the overall water permeation flux.

(1) $$J_{W}=A\cdot \left(\frac{\pi_{Dbulk}\cdot e^{-J_{w}{}^{*}K}-\pi_{Fbulk}\cdot e^{\frac{J_{W}}{k}}}{1-\frac{B}{J_{W}}\cdot (e^{-J_{w}\cdot K}-e^{\frac{J_{W}}{k}})}+P_{F}-P_{D}\right)$$

where *J_w_* is the water flux, *A* is the membrane-specific pure water permeability, *B* is the membrane-specific solute permeability, *π_Fbulk_* and *π_Dbulk_* are the osmotic pressure of the feed and the draw solutions, respectively, *P_F_* and *P_D_* are hydraulic pressure in the feed and the draw side, respectively, *k* is the mass transfer coefficient in the feed channel, and *K* is the resistance to solute diffusion (s·m^−1^) and defined as Equation (2):

(2) $$K=\frac{t_{s}\cdot \tau }{D\cdot \varepsilon }=\frac{S}{D}$$

where *t_s_* is the thickness (m), *τ* is the tortuosity and *ε* is the porosity of the support layer and *D* is the self-diffusion coefficient of the solute. These parameters are characteristics of the membrane, and the *S* factor in which they are combined is commonly referred to as the structural parameter [51]. Membrane specific values were measured to be 10 L·m^−2^·h^−1^·bar^−1^, 2 × 10^−6^ m·s^−1^ and 2 × 10^−4^ m for *A*, *B* and *S* respectively. Typical values found in the literature are slightly lower for *A* (6–9 L·m^−2^·h^−1^·bar^−1^) and higher for *B* (2.5–3.3 × 10^−6^ m·s^−1^) and *S* (2.15–2.7 × 10^−4^ m) [14,52,53,54], and may be due to the variability of the sample received.

## 3. Results

### 3.1. Initial Test

**Figure 4:**
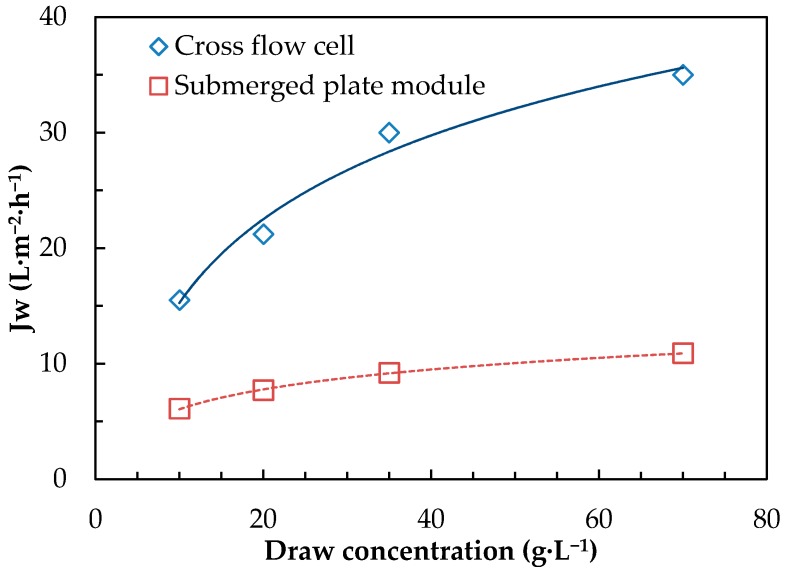
Comparative water permeation flux (*Jw* in L·m^−2^·h^−1^) as a function of SW draw solution concentration (g·L^−1^) using DI water as feed for the CFC and submerged plate module (a pumping flow rate of 0.3 L·min^−1^ for both setups, no stirring for the submerged FO module).

A U-shape FO submerged plate was first tested using DI water as feed for a range of draw solution concentrations varying from 10 to 70 g·L^−1^. The resulting water permeation fluxes were compared with those from membrane coupon cut from this plate but operated in the CFC setup (Figure 4).

The submerged plate module featured a permeation flux about 3 times lower than the one in the CFC for the whole range of draw concentration tested. Such severe loss of performance emphasizes the need of better understanding of mass transfer limitation in submerged FO systems. If both systems were operated with similar membranes, similar feed and draw solutions and similar draw solution flow rates, they also featured many distinct critical operating parameters that can influence system efficiency from both the feed and the draw sides, such as:

- positive pressure pumping (feed and draw) for CFC versus negative pressure pumping for submerged plates (draw)
- straight draw channel for CFC versus U-shape design for plates
- different CFV
- optimized feed cross flow channel for CFC versus an unstirred submerged system for plates

The impact of each parameters mentioned above was systematically assessed in the following sections.

### 3.2. Draw Side Limitations

#### 3.2.1. Positive (Pulsion) vs. Negative (Vacuum) Draw Circulation (Using the CFC Setup)

**Figure 5:**
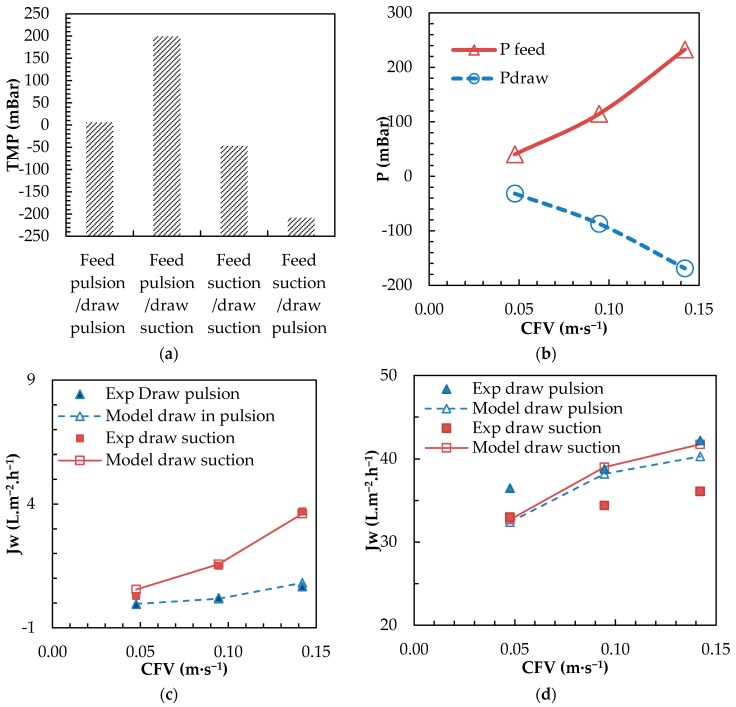
Tests with CFC cells. (**a**) Impact of feed and draw pumping modes on TMP (CFV of 0.9 m·s^−1^ for both feed and draw channels, DI as feed and draw), (**b**) impact of feed and draw CFV on respective feed and draw channel pressure (feed operated in pulsion, draw in suction, DI as feed and draw) and comparison of experimental and modelled water flux values (*Jw*) for different CFVs and operation of draw either in suction or pulsion with (**c**) DI as feed and draw and (**d**) DI as feed and 35 g L^−1^ sea salts as draw.

Submerged MBR plate modules are typically operated using depression by negative pressure pumping (suction) in the permeate line, as it is the driving force for water permeation. Applying positive pressure on the permeate line is sometimes used as backwashing typically in hollow fiber configurations, but cannot be applied to plate modules due to practical and mechanical limitations (the membrane inflates and may break). In submerged osmotic processes (using plate and frame), pressure is not the driving force, but the draw has to be circulated between the plate and the membrane. Thus, as for submerged MBRs, circulating the draw solution in positive pressure (pulsion) is not desired due to similar membrane mechanical limitations (the membrane inflates and may break). Pumping operation in suction is unusual in osmotic processes normally operated in the cross flow mode with the positive pressure pumping mode (pulsion) [14]. Therefore, the impact of operating feed and/or draw channel in pulsion and or suction was evaluated in terms of pressure balance, permeation flux and for different CFVs using a CFC (Figure 5). Experimental flux results were compared to modelled flux obtained from Equation (1), which included pressure data obtained experimentally (Figure 5c,d).

It was first observed that, depending on how feed and draw channels are operated in osmotic processes, significant differences of transmembrane pressure (TMP) can be expected (Figure 5a). Typically, if feed and draw channel are operated with a similar pumping mode, no/low TMP is observed since pressure is balanced on the two sides of the membrane. On the contrary, when feed and draw were operated in an opposite pumping mode, significant TMP was observed (±200 mbar at 0.1 m s^−1^ CFV). TMP was dependent from the CFV with both feed and draw solutions (Figure 5b), and consequently, even higher TMP was obtained (±400 mbar at 0.15 m·s^−1^, i.e., P_feed_ = +233 and P_draw_ = −169 mbar). With operation in pulsion in the feed channel and suction in the draw, the TMP acts towards increase of the permeation flux (Equation (1)) and can be assimilated to pressure assisted osmosis [50,55] or vacuum assisted osmosis (VAO) in this particular case.

The interest of VAO was then assessed for different CFVs, and experimental values with model data obtained using Equation 1, with DI water as draw (pressure driven permeation only (Figure 5c) and with 35 g·L^−1^ SW as draw were compared (Figure 5d). Good fitting of modelled and experimental water fluxes were observed when DI water was used on the draw side (Figure 5c). In addition, higher permeation flux was observed with DI draw operation in the suction mode, confirming the interest of VAO towards flux improvement, especially when a high permeability FO membrane is used. CFV, as a result of increased TMP, also proved to have a non-negligible effect towards flux improvement. However, when operated with SW as draw, the benefit of VAO was no longer observed experimentally, since in all cases, higher fluxes were achieved when draw was operated in pulsion (Figure 5d). This is not in line with expectation and modelled values and can be attributed to imperfect hydrodynamics in the draw line when operating in suction. It could be hypothesized that membrane displacement occurs as a consequence of TMP creating dead zones with intense concentration polarization; computational fluid dynamics (CFD) modelling may help to confirm that hypothesis [17,56]. Moreover, modelled values for both operating modes were closed, indicating that TMP had only a moderate impact when operating with SW as draw as the system relies mainly on the osmotic driving force. The improvement of flux with CFV, therefore, cannot only be attributed to increased TMP, but is mainly the consequence of improved turbulences (higher *k* values) limiting ECP on the feed side.

#### 3.2.2. Draw Channel Design (Submerged Plate)

**Figure 6:**
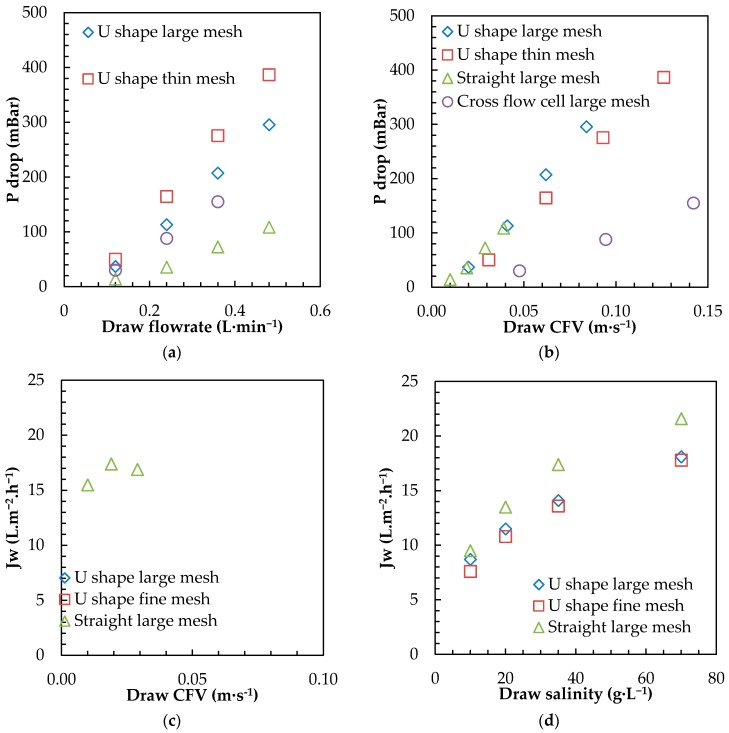
Impact of (**a**) draw pumping flow rate and (**b**) CFV on pressure drop in tested P&F modules (DI water as feed and draw solutions, draw pumping operated in suction). Water permeation flux as a function of (**c**) draw CFV and (**d**) draw salinity for the 3 tested P&F modules (DI water as feed 35 g·L^−1^ SW as draw, draw pumping operated in suction, feed side under mechanical stirring).

As already pointed out in a recent study on the OMBR, although osmotic processes rely on osmotic gradient, significant energy for draw circulation can be required, depending on module design and offsetting initial energy benefits [46]. To get more insight of upscaling and importance of module design, further tests were achieved with 3 designed plates and compared with CFC in terms of pressure drop and flux for different draw flow rates and salinities (Figure 6).

For a similar draw recirculation flow rate, U-shape plates featured much higher pressure drop than the CFC or straight channel modules (Figure 6a). When plotting the pressure drop as a function of the CFV (Figure 6b), all modules presented similar slopes, indicating that differences observed in Figure 6a were the consequences of the module cross section; i.e., smaller channels width for U shape modules and thinner channel for thin mesh spacers. However, straight channel design appears to be the optimized design, as it allows for the lowest pressure drop (and lower energy consumption), due to a higher cross section (very low CFV) and a better repartition of the draw flow. All plates design had significantly higher pressure drops than the CFC for similar flow rates, which indicated either other hydrodynamics plate limitations (inlet/outlet connections) or some preferential passage may occur in the CFC with an overall channel thickness larger than the spacer itself. CFD modelling will help to further understand and improve current design limitations [17].

For the first time, fluxes above 15 L·m^−2^·h^−1^ were obtained with all submerged modules and SW as the draw solution (Figure 6c), showing the general improvement with the use of high permeability FO TFC membrane and the design developed in this study largely outperforms all former configurations tested [29,46]. A flux as high as 21.6 L·m^−2^·h^−1^ with 70 g·L^−1^ SW as a draw solution (Figure 6d) has been reached with the straight channel module, paving the way for higher competitiveness of submerged osmotic processes. The two U-shape modules featured very similar water permeation fluxes for the set of flow rate (Figure 6c) and draw salinity (Figure 6d), indicating that draw channel spacer did not have a significant impact on the permeation flux. The straight channel design demonstrated systematically higher permeation (2–4 L·m^−2^·h^−1^) flux regardless of the flow rate and the draw salinity used. The difference between U-shape and straight channel modules confirmed the limitation of U-shape design already pointed out in other FO modules with dead zones, leading to areas with intense concentration polarization and very low local fluxes [57]. In addition, very interestingly, the straight channel module proved to allow for high flux despite lower CFV (and lower pressure drop), and therefore, is beneficial for a significant improvement in module design.

### 3.3. Feed (Submerged) Side Limitations

#### 3.3.1. External Concentration Polarization Evidence (Stirred Versus Unstirred Feed Tank)

**Figure 7:**
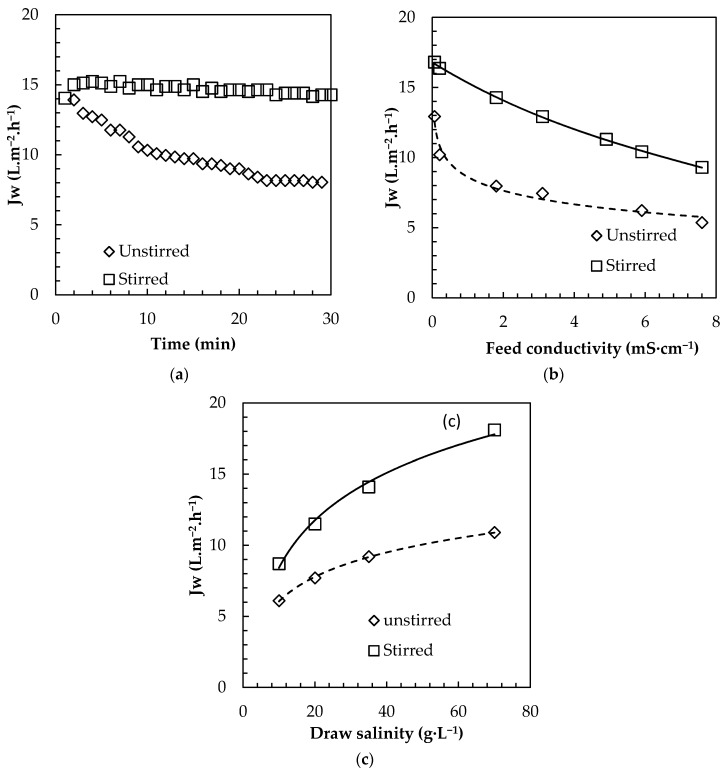
Impact of stirring on (**a**) instantaneous water flux, and average permeation flux (during 30 min) (**b**) for different feed conductivities (**c**) and different draw salinities (DI water as feed, 35 g·L^−1^ SW as draw solution, U shape large mesh plate).

The impact of ECP was then evaluated by submerging modules in feed solutions with different salinities and by operating the system under no stirring or with intense mixing (Figure 7).

When comparing water flux over the first 30 min of filtration with and without mixing, very distinct behavior was observed (Figure 7a). Initial fluxes were very similar when filtration started, around 15 L·m^−2^·h^−1^, but flux was maintained constant under mixing conditions while a sharp decline down to 8 L·m^−2^·h^−1^ occurred in unstirred system, confirming a very intense and quick ECP buildup. Such ECP happened even when DI water was used as feed and thus indicates that observed ECP was the consequence of reverse salt diffusion (RSD). Feed salinity proved to have a crucial impact also on the permeation flux (Figure 7b). In the case of stirred system where ECP is limited, flux decrease with increased conductivity is a direct consequence of the loss of driving force (salinity gradient). For the unstirred system, water flux dropped significantly and was even more pronounced when salts are presents in the feed indicating that feed salinity further enhances ECP (Figure 7b).

The impact of mixing on ECP was further confirmed for different draw salinities (Figure 7c) with obtained water flux in unstirred system at least 30% lower than for stirred sytem. Typically, in unstirred system, the expected benefit of increasing the salinity gradient towards flux enhancement is partly lost by the enhanced ECP (due to higher initial flux) and higher RSD (due to higher draw concentrations) [12]. Under unstirred conditions, ECP is the predominant phenomenon and avoids noticing a significant difference of positive/negative impact of other process parameters (temperature, module design, draw solution). As for FO membranes characterization [58], it emphasizes the importance of setup design to evaluate module performances; using stirred conditions is strongly advised for submerged osmotic systems.

#### 3.3.2. External Concentration Polarization Mitigation with Air Scouring and Feed Tank Recirculation

Intense stirring proved to be efficient to mitigate ECP but is highly energy intensive and not always adapted to (potential) submerged FO applications (1) due to the high shearing it produces and (2) the difficulty to implement it in a membrane stack. Other alternatives to promote turbulences, such as air scouring and recirculation, were then evaluated.

**Figure 8:**
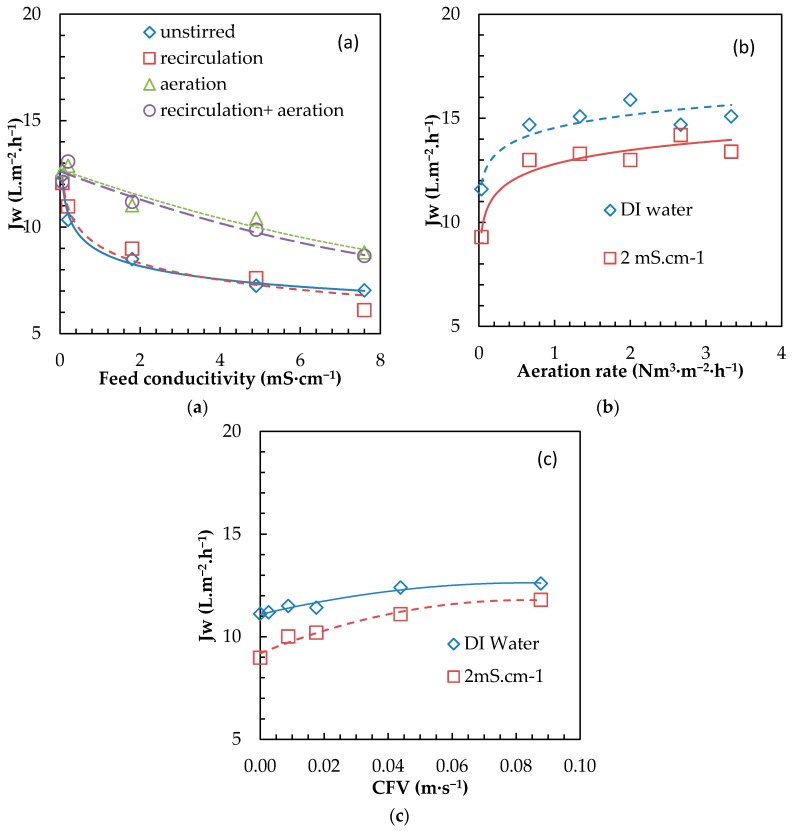
Impact of (**a**) aeration and recirculation, (**b**) intensity or aeration and (**c**) CFV of recirculation on water permeation flux (35 g·L^−1^ SW as the draw solution, U-shape large-mesh plate).

At first, similar operation parameters (recirculation and aeration) as applied in our recent MBR/OMBR studies were tested and evaluated (Figure 8) for different feed conductivities (Figure 8a). No significant flux improvement was observed when a typical MBR recirculation rate was applied, but higher fluxes were achieved with aeration (only or combined with recirculation). A similar trend, as shown in Figure 7b, indicates that aeration is a promising strategy to overcome ECP. As such, quite interestingly, in submerged FO systems, the role of aeration is triple since it can not only bring oxygen to the bioreactor and help to mitigate fouling (as in MBR), but also provide an efficient method to tackle ECP. In processes where air scouring is not required, other gases could be envisioned, such as nitrogen or biogas for Anaerobic OMBRs [39].

The aeration rate was then evaluated for 2 types of feed water (Figure 8b). In both cases, the impact of aeration (with/without) was clearly confirmed with an immediate increase of 40% in permeation flux even for the lowest aeration rate tested (0.67 m^3^·m^−2^·min^−1^). Above that value, minimal increase of permeation flux was observed, indicating that the typical aeration rate as commonly applied in MBR (0.6–1.5 Nm^3^·m^−2^·min^−1^) [34] is sufficient (for ECP mitigation purposes). That can be explained by the fact that optimized mixing is already reached. In addition, with intense bubbling, air present at the membrane surface instead of water can decrease surface contact between water and the membrane, resulting in degradation of permeation flux. As for the submerged MBR, further work dedicated to the impact of bubble size, as well as calculation of aeration induced CFV, will help to further improve understanding and operation of submerged FO system for ECP mitigation [59,60].

CFV also proved to have an impact on permeation flux (Figure 8c) but to a lesser extent than the aeration rate. The optimum increase was limited to 32% (from 8.9 to 11.8 L·m^−2^·h^−1^) and observed for saline feed water and the highest CFV tested (0.09 m·s^−1^). As such, ECP mitigation through CFV proved to be efficient only in the case of the high recirculation rate; such a configuration is not of application in OMBR but can be found, for example, in microalgae raceway ponds. Aeration appeared to be the preferred option for ECP mitigation, but high CFV can be applied if dedicated submerged OMBR designs are developed. Other options like mechanical shaking are still to be tested as other alternatives [61].

## 4. Conclusions

This study demonstrated that the knowledge learnt from MBR and existing cross flow FO modules cannot be directly applied to submerged FO configurations, where the draw recirculation rate, module design and ECP mitigation are three key issues largely affecting water permeation fluxes. High pressure drops and dead zones occurring in classical U-shape FO draw channel strongly disadvantage this design; straight channel design proved to be more effective and further improvement can be realized. Development and evaluation of submerged hollow fiber modules still needs to be explored. ECP is also a crucial element in the submerged FO process since mixing of the feed solution is not as optimized as in the cross flow module unless applying intense stirring. Among the mitigation techniques tested, air scouring proved to be most efficient. Further work is required to develop ECP mitigating strategies adapted to application specificities and with regards to combined/synergetic effects with fouling mitigation.

## Figures and Tables

**Table 1:** CFV associated with a range of pump flow rate tested for CFC (feed and draw) and submerged modules (draw).

Pump Flow Rate	L·min^−1^	0.12	0.24	0.36	0.48
CFC (feed and draw)	m·s^−1^	0.05	0.09	0.14	/
U shape large mesh	m·s^−1^	0.02	0.04	0.06	0.08
U shape fine mesh	m·s^−1^	0.031	0.062	0.093	0.126
Straight large mesh	m·s^−1^	0.01	0.019	0.029	0.039
